# Supplementary figures and images for: Functional Connectivity Predictors and Mechanisms of Symptom Change in Functional Neurological Disorder
Source: medRxiv. 2026 Jan 30:2026.01.27.26344860. Preprint. [Version 1] doi: 10.64898/2026.01.27.26344860 (PMC12870675; doi:10.64898/2026.01.27.26344860)

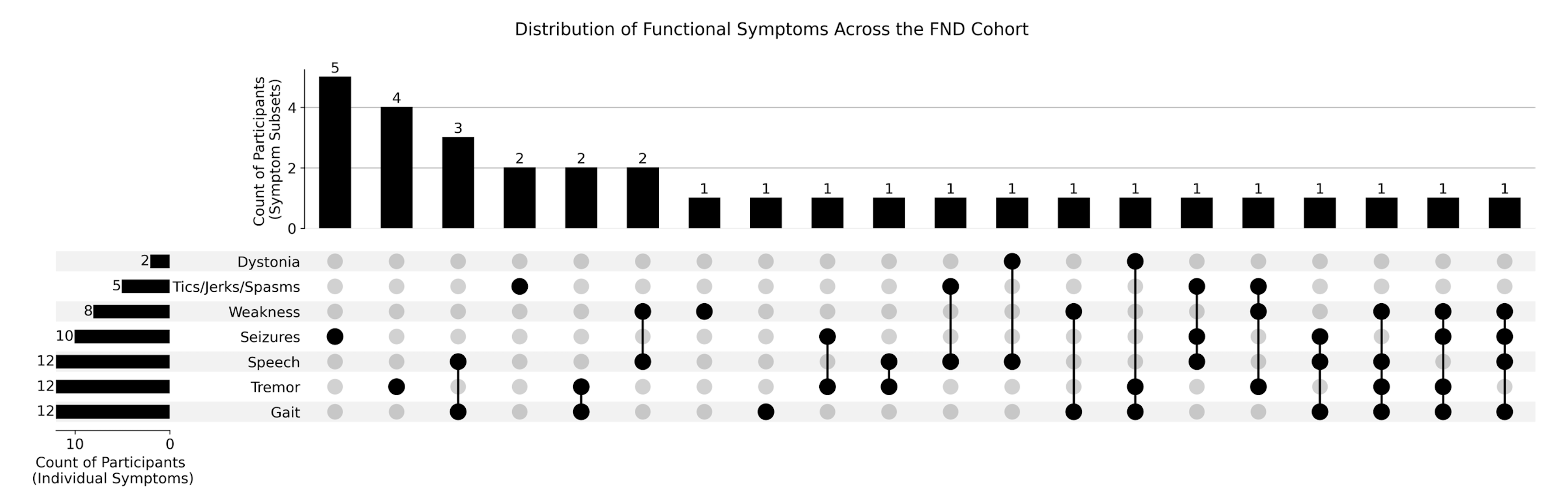

Supplement: Supplement 2 [file media-2.tif]

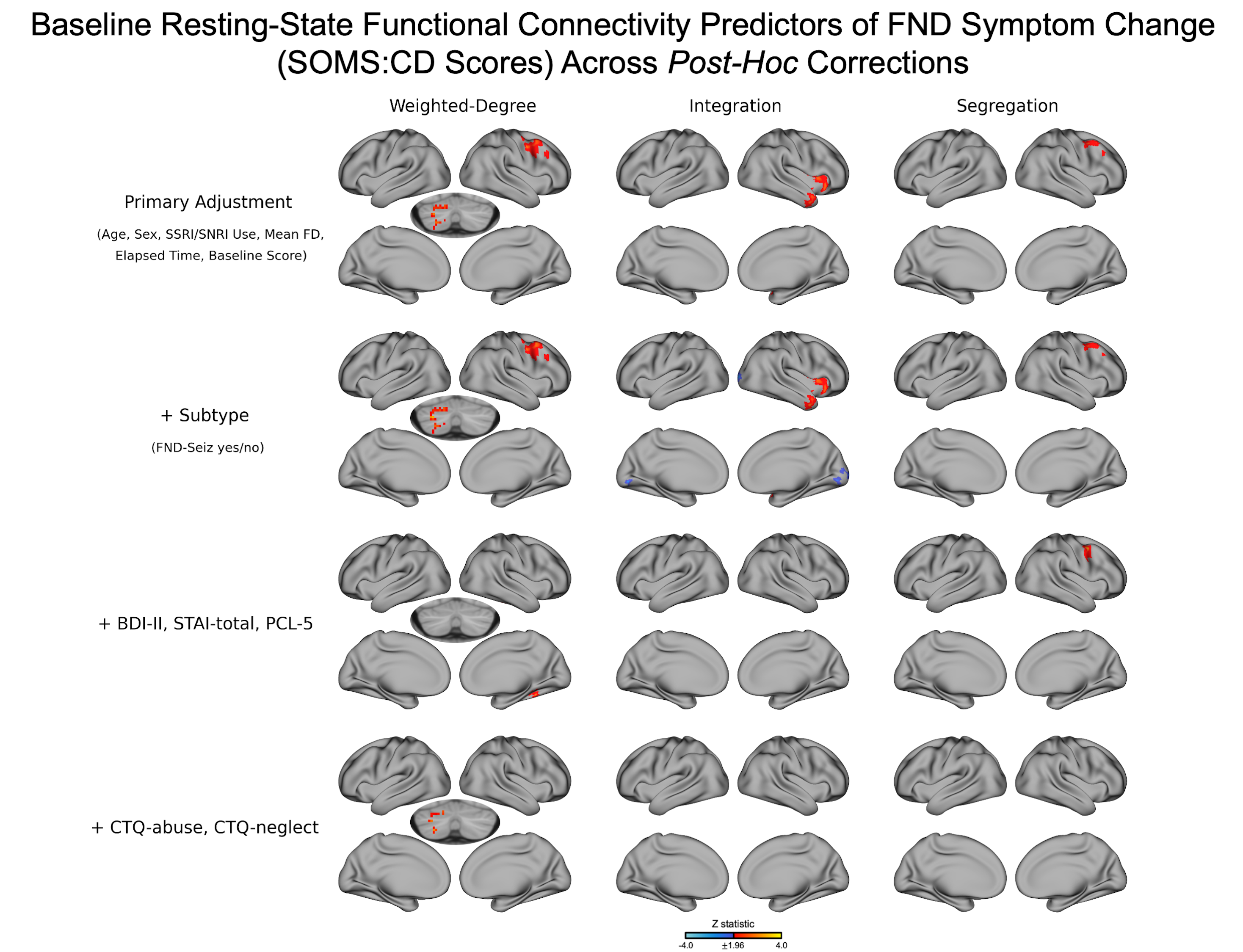

Supplement: Supplement 3 [file media-3.tif]

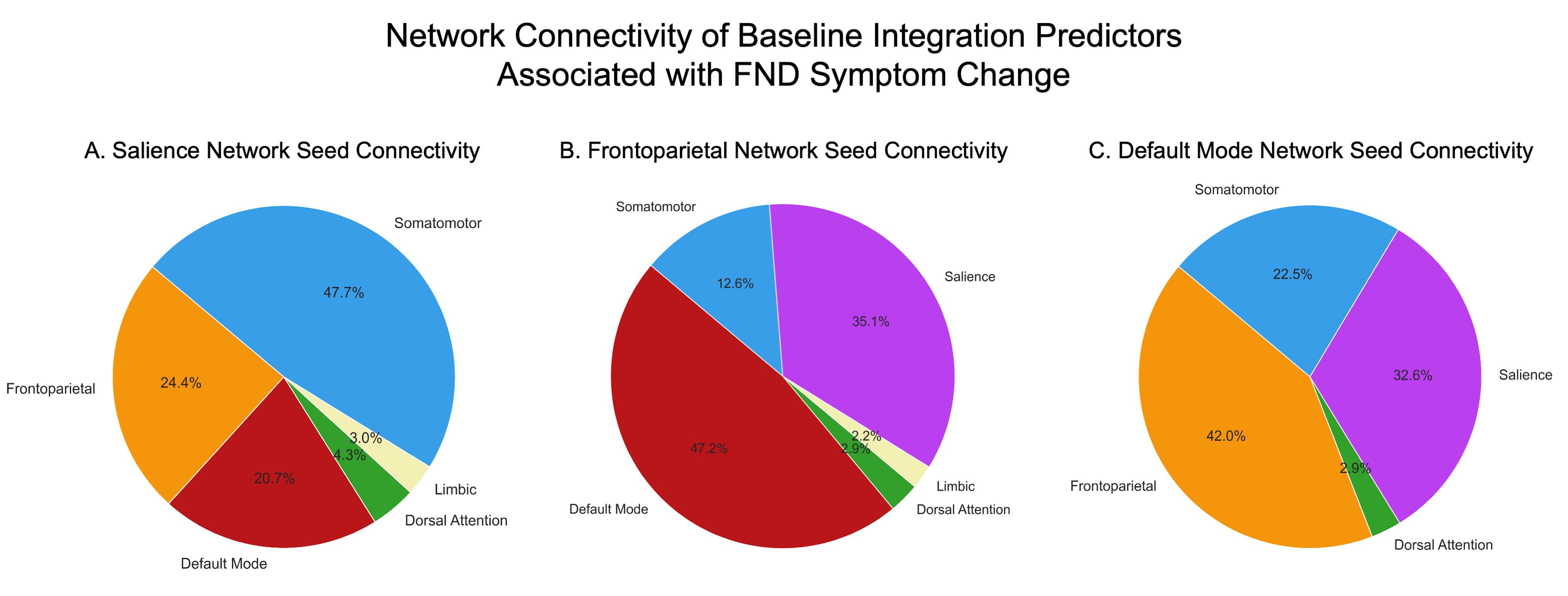

Supplement: Supplement 4 [file media-4.tif]

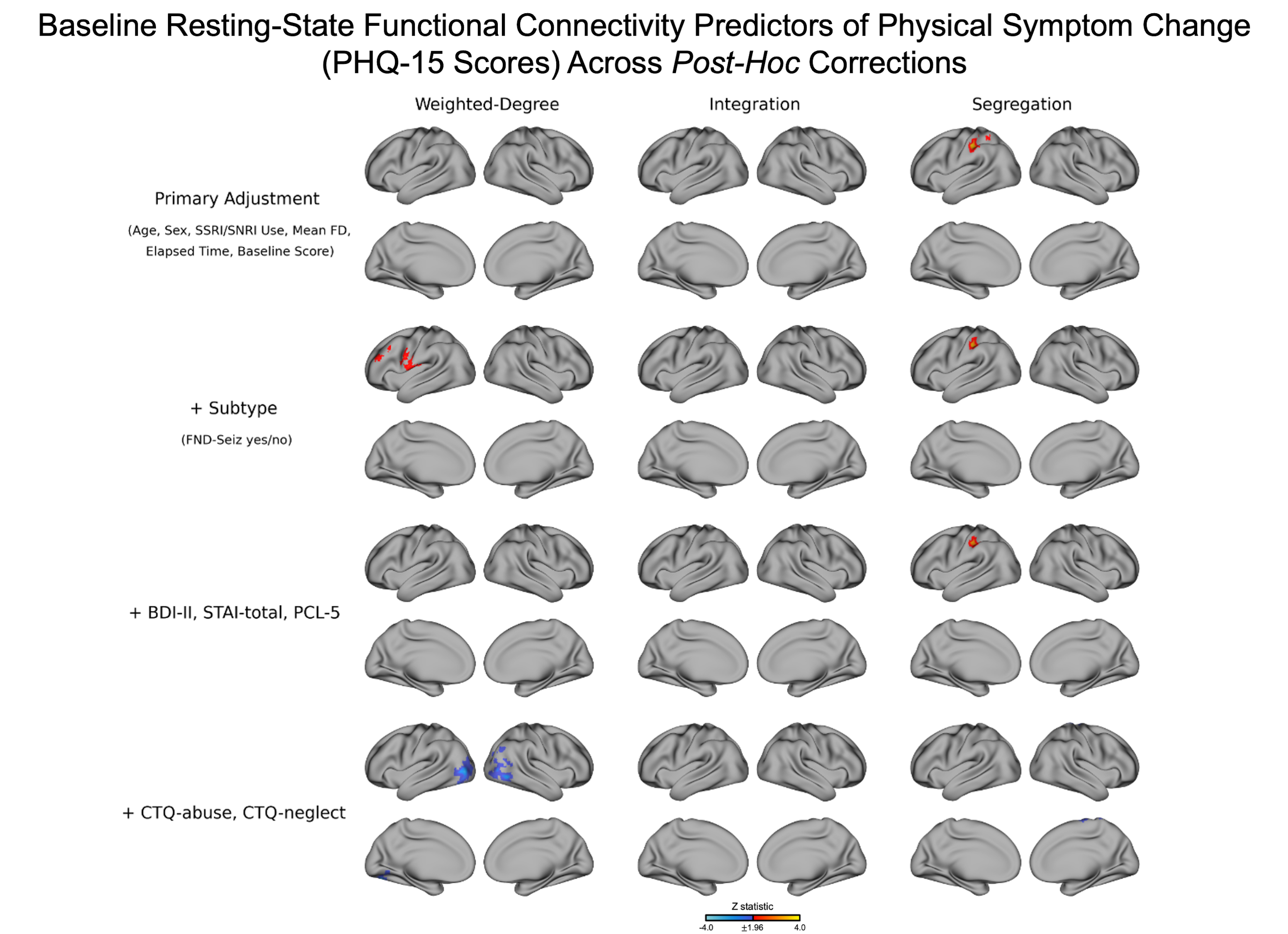

Supplement: Supplement 5 [file media-5.tif]

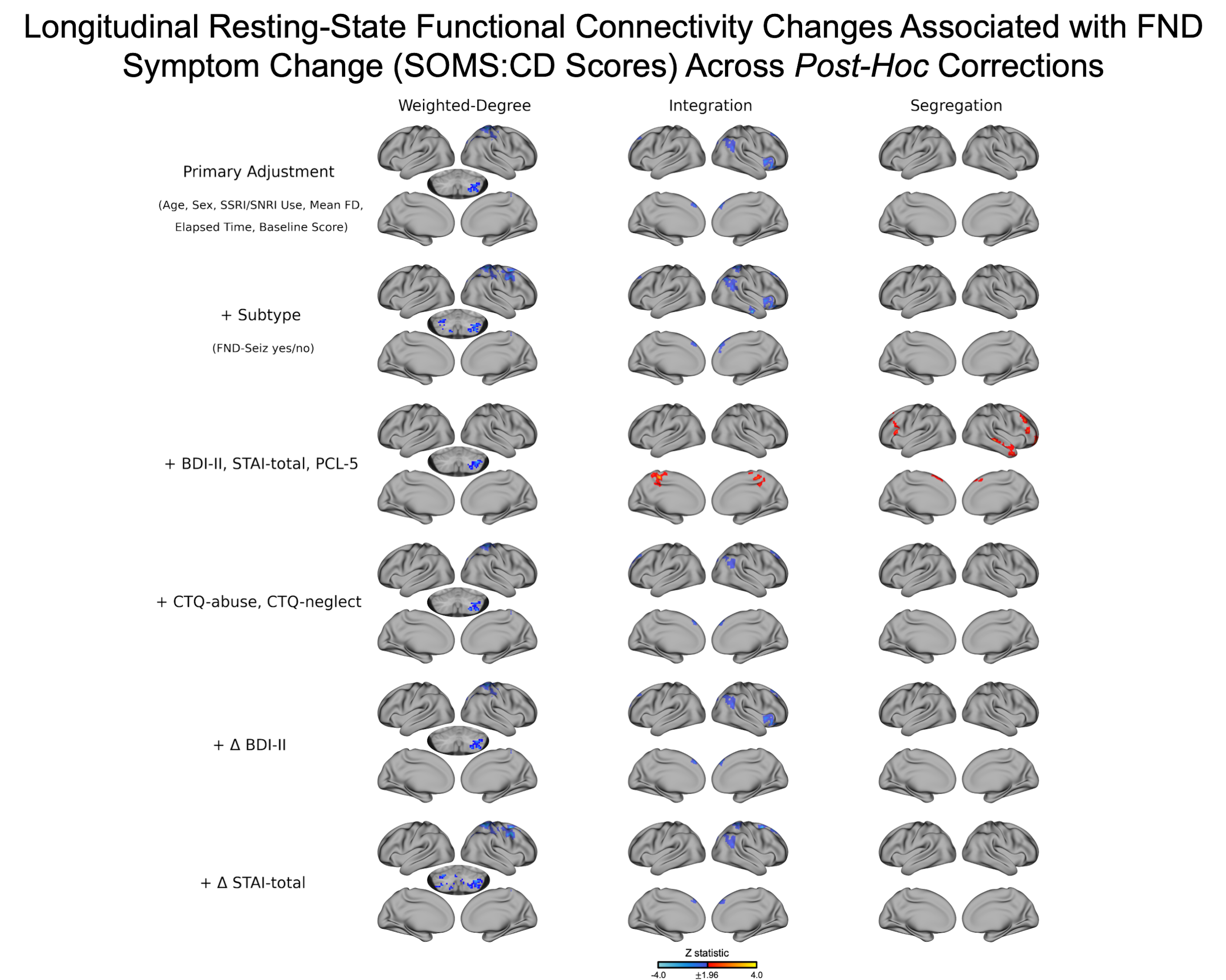

Supplement: Supplement 6 [file media-6.tif]

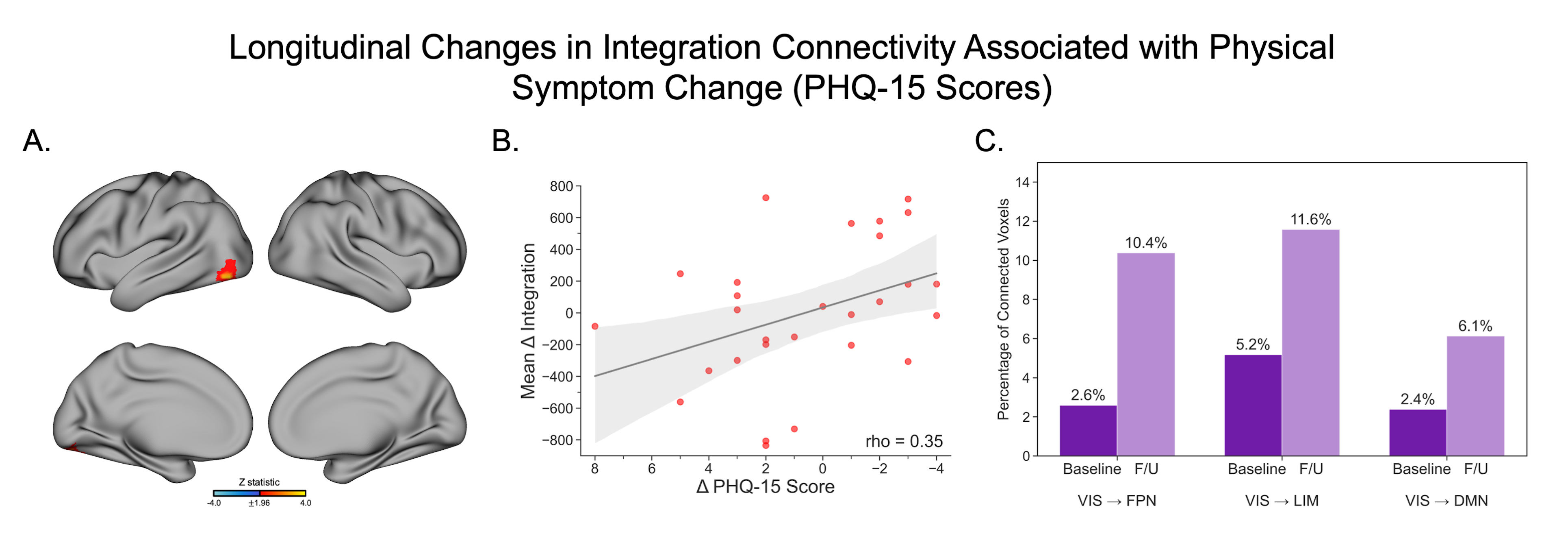

Supplement: Supplement 7 [file media-7.tif]

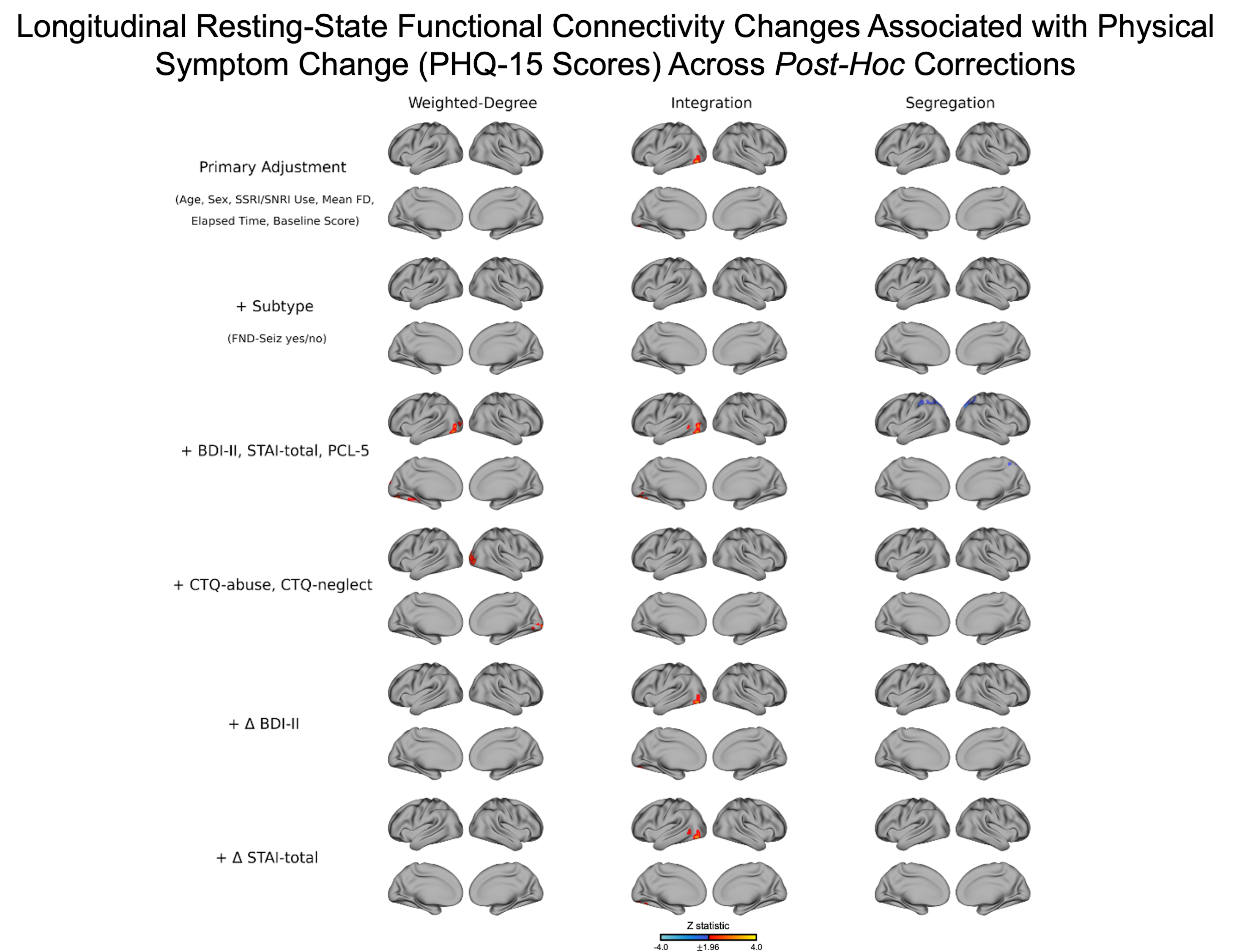

Supplement: Supplement 8 [file media-8.tif]
